# Supplementary material for: Comparative Genomic Analysis of SAUR Gene Family, Cloning and Functional Characterization of Two Genes (PbrSAUR13 and PbrSAUR52) in Pyrus bretschneideri
Source: Int J Mol Sci. 2022 Jun 24;23(13):7054. doi: 10.3390/ijms23137054 (PMC9266570; doi:10.3390/ijms23137054)
Supplement: Supplementary file 1 [file ijms-23-07054-s001.zip › ijms-1775990 Figures S1 and S2.pdf]

Motif 1

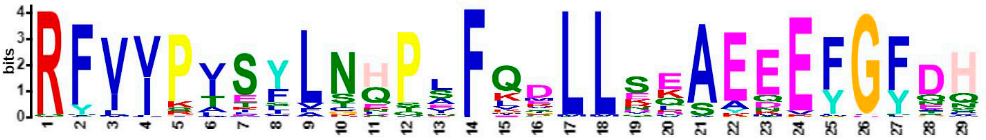

Motif 2

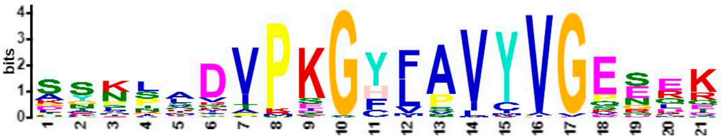

Motif 3

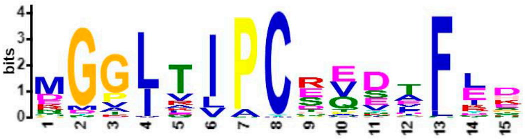

Motif 4

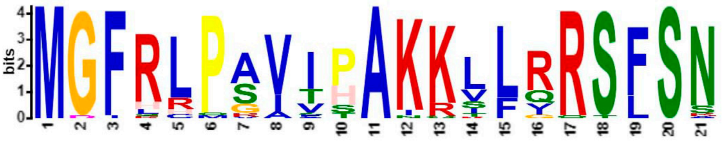

Motif 5

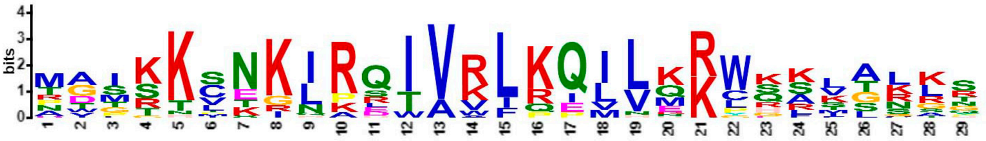

Figure S1. Conserved motif composition

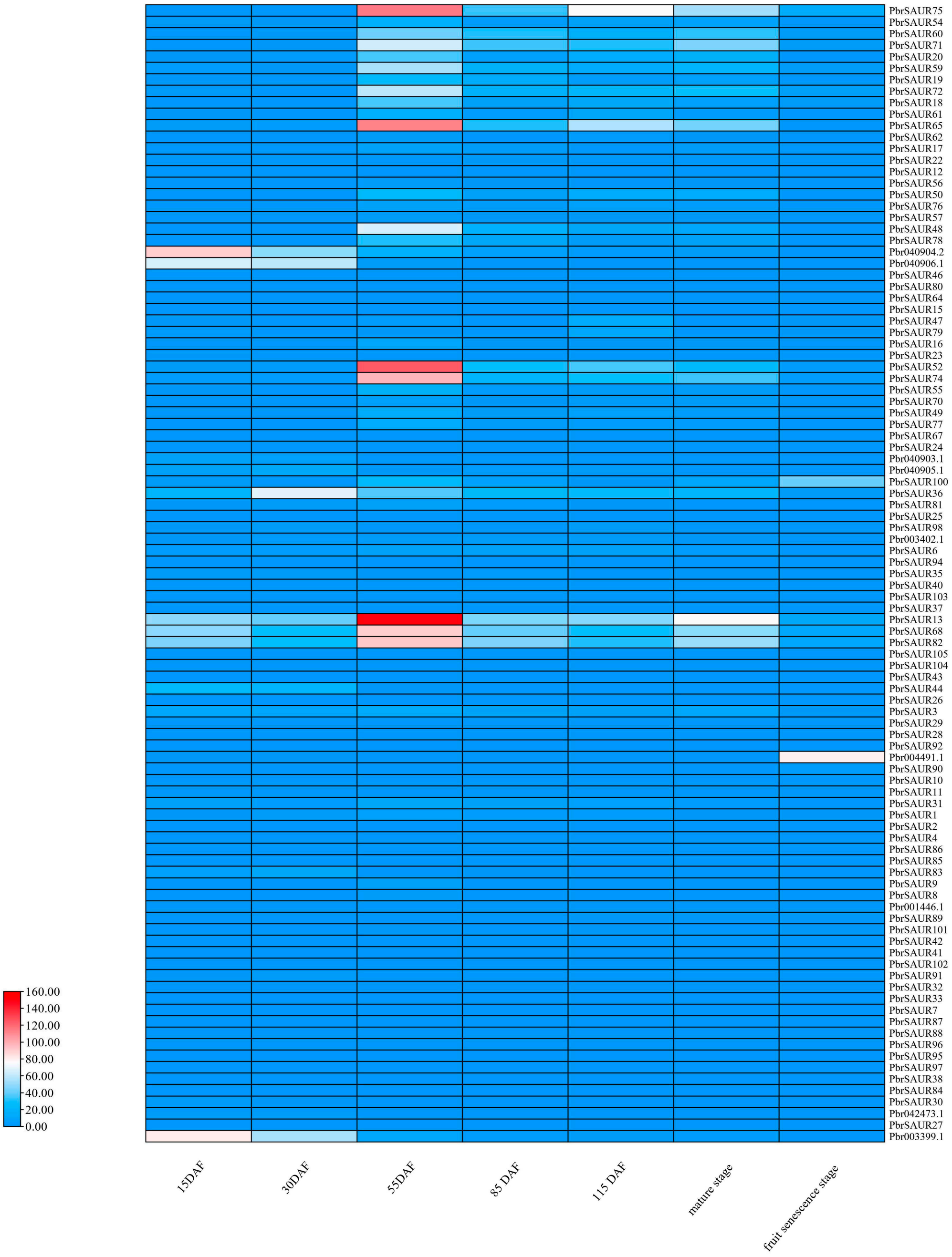

Figure S2. Expression patterns of *PbrSAUR* gene family at different developmental stages
